# PPARgene: A Database of Experimentally Verified and Computationally Predicted PPAR Target Genes

**DOI:** 10.1155/2016/6042162

**Published:** 2016-04-11

**Authors:** Li Fang, Man Zhang, Yanhui Li, Yan Liu, Qinghua Cui, Nanping Wang

**Affiliations:** ^1^Institute of Cardiovascular Sciences, Peking University Health Science Center, Beijing 100191, China; ^2^Department of Biomedical Informatics, Peking University Health Science Center, Beijing 100191, China; ^3^The Advanced Institute for Medical Sciences, Dalian Medical University, Dalian 116044, China

## Abstract

The peroxisome proliferator-activated receptors (PPARs) are ligand-activated transcription factors of the nuclear receptor superfamily. Upon ligand binding, PPARs activate target gene transcription and regulate a variety of important physiological processes such as lipid metabolism, inflammation, and wound healing. Here, we describe the first database of PPAR target genes, PPARgene. Among the 225 experimentally verified PPAR target genes, 83 are for PPAR*α*, 83 are for PPAR*β*/*δ*, and 104 are for PPAR*γ*. Detailed information including tissue types, species, and reference PubMed IDs was also provided. In addition, we developed a machine learning method to predict novel PPAR target genes by integrating* in silico* PPAR-responsive element (PPRE) analysis with high throughput gene expression data. Fivefold cross validation showed that the performance of this prediction method was significantly improved compared to the* in silico* PPRE analysis method. The prediction tool is also implemented in the PPARgene database.

## 1. Introduction

Peroxisome proliferator-activated receptors (PPARs) are ligand-activated transcription factors that belong to the superfamily of nuclear receptors. PPARs form heterodimers with a retinoid X receptor (RXR) and control gene expression by binding to specific PPAR-responsive elements (PPREs) on target gene promoters [1]. PPARs play critical roles in the regulation of lipid and glucose metabolism, inflammation, wound healing, and many other pathophysiological processes [2–5]. Synthetic PPAR ligands, such as fibrates and thiazolidinediones, are used for clinical treatment of dyslipidemia and type 2 diabetes, respectively [6].

Extensive studies have demonstrated a variety of target genes regulated by the individual PPAR subtype. Therefore, building a database with a comprehensive collection of the previously verified PPAR target genes for each subtype will be helpful for PPAR research. In this study, we first established a database of PPAR target genes, PPARgene. Experimentally verified PPAR target genes were manually curated and detailed information including PPAR subtype, tissue types, species, and reference PubMed IDs was provided.

Recently, the application of high throughput technologies such as microarray has generated a number of PPAR-induced gene expression data sets, which are freely available in public database. By integrating* in silico* PPRE analysis with high throughput gene expression data, we developed a machine learning method to predict novel PPAR target genes. The prediction tool is also implemented in the PPARgene database (http://www.ppargene.org/).

## 2. Methods

### 2.1. Data Collection

#### 2.1.1. Collection of Experimentally Verified PPAR Target Genes

PPAR-related publications were acquired from PubMed database using the key words “PPAR”, “PPAR alpha”, “PPAR beta”, “PPAR delta”, “PPAR gamma”, or “peroxisome proliferator” (review articles were excluded). We then curated the data manually and retrieved the PPAR target genes if experimental evidence for gene regulation (at mRNA and/or protein levels) and functional PPRE (reporter assay and/or DNA-binding assays) were both reported.

#### 2.1.2. Collection of PPAR-Relevant Microarray Data Sets

PPAR-relevant microarray data sets were acquired by searching the GEO database [7] using the key words “PPAR”, “PPAR alpha”, “PPAR beta”, “PPAR delta”, “PPAR gamma”, or “peroxisome proliferator”. We manually curated 22 data sets in which PPARs were activated or overexpressed.

### 2.2. Feature Extraction

#### 2.2.1. High Throughput Evidence (HTE)

To obtain the high throughput experimental evidence supporting PPAR target gene interactions, we collected microarrays in which PPARs were activated or overexpressed. Raw data of collected microarrays were processed using the R-packages Bioconductor [8]. The HTE value of a gene was defined as total number of data sets divided by number of data sets in which this gene was upregulated (log_2_ fold change > 0.5).

#### 2.2.2. PPRE Score (PS)

Reference genome of mouse (GRCm38) and rat (Rnor_6.0) was downloaded from NCBI. According to previous studies [9–12], PPREs were located within 5 kb upstream or downstream of the transcription start site (TSS) in most cases. Therefore, we extracted −5 kb~+5 kb TSS flanking sequences from the reference genome for all mouse and rat genes identifiable by Entrez Gene ID according to the genomic coordinates.

Potential PPREs were scanned* in silico* using the position weight matrix (PWM) model, which was widely used to describe cis-regulatory elements [13, 14]. Since the three subtypes of PPARs bind to a common core consensus sequence, we did not distinguish the difference of binding site among subtypes and used the position frequency matrix (PFM) of PPAR*γ*-RXR*α* heterodimer retrieved from JASPAR database (ID: MA0065.2) [15] to compute the PWM of PPRE. The PWM was computed as described previously [16]. Briefly, we calculated the PWM value as

(1) $$\begin{array}{c} W_{b,i}=\log_{2}\frac{p\left(b,i\right)}{p\left(b\right)}, \end{array}$$

where *W*_*b*,*i*_ is PWM value of base *b* in position *i*, *p*(*b*) is background probability of base *b* in the genome, and *p*(*b*, *i*) is probability of base *b* in position *i*. Pseudocount values (square root of the number of sites) were added to each base in each position to smoothen the small sample effects. The PWM score for a putative sequence was calculated as sum of the PWM values for each nucleotide in the sequence. For each gene identifiable by Entrez Gene ID in the mouse genome, we scanned putative PPREs from the TSS flanking sequences in both strands at a PWM score cut-off of 4.56 (70% relative to top PWM score) initially. The PS value of a gene was defined as the highest PWM score of all PPREs identified in this gene.

#### 2.2.3. Conserved PPRE Score (CPS)

Evolutionary conservation has been used as an effective filter for improving specificity in regulatory motif recognition [17–19]. We performed comparative genomic analysis to identify conserved PPREs. Pairs of orthologous genes in mouse and rat were retrieved from NCBI HomoloGene database. TSS flanking sequences (−5 kb~+5 kb) of the orthologous gene pairs were aligned using megaBLAST with default parameters (word size = 28, reward = 1, mismatch penalty = −2, gap opening penalty = 0, and gap extension penalty = 2.5) [20, 21]. Alignments less than 50 bp or with an *E*-value > 0.001 were discarded. For each orthologous gene, we scanned putative PPREs from the TSS flanking sequences at a PWM score cut-off of 4.56. A pair of putative PPREs was identified as conserved PPRE if they were matched in the pairwise alignments. The CPS value of a gene was defined as the highest PWM score of all conserved PPREs identified in this gene.

### 2.3. Model Training and Evaluation

#### 2.3.1. Training Sets for the Prediction Model

Experimentally verified target genes collected in the PPARgene database were defined as positive training samples. However, it would be difficult to prove that a gene is not a target gene of PPARs in any conditions. Thus, we obtain negative training samples by randomly choosing equal number of genes from the background data set, which contained all protein coding genes excluding the positive samples. To avoid sampling bias, we sampled the negative data set 100 times and then combined each negative data set with the positive data set to train the classifier.

#### 2.3.2. Logistic Regression Classifier

We employed the binomial logistical regression model to predict PPAR target genes. All mouse protein coding genes with a HomoloGene database ID were classified according to a combination of the features described above. Let *p*_*i*_ be the probability that the *i*th gene is a PPAR target gene and let 1 − *p*_*i*_ be the probability that it is not. The logistic regression model is

(2) $$\begin{array}{c} \log\frac{p_{i}}{1-p_{i}}=\beta_{0}+\overset{M}{\underset{j=1}{\sum }}\beta_{j}X_{ij}, \end{array}$$

where *β*_*j*_ is the regression coefficient of the feature *X*_*ij*_. The logistic regression model was implemented using the generalized linear model (GLM) function in R [22].

#### 2.3.3. Performance Evaluation

We used 5-fold cross validation to evaluate the performance of the logistic regression model. In each round, 20% of the samples were left out as the test data and the remaining were the training data. Precision, recall, and *F*1 score were used to evaluate the performance of the classifier. Precision, recall, and *F*1 were calculated as

(3) Precision=TPTP+FP,Recall=TPTP+FN,F1=2  precision×recallprecision+recall,

where TP is the number of true positives, FP is the number of false positives, and FN is the number of false negatives. We also calculated AUC, the area under the receiver operating characteristic (ROC) curve, using ROCR package [23]. Because negative data sets were obtained by 100 random samplings, the medians of precisions, recalls, *F*1s, and AUCs of the 100 training results were used.

### 2.4. Web Server

All data were organized using MySQL, an open-source relational database management system. The website was presented using PHP. The PPARgene database is freely available at http://www.ppargene.org/.

## 3. Results and Discussion

### 3.1. Experimentally Verified PPAR Target Genes

In this study, we developed a database for PPAR target genes. We curated PPAR target genes manually from 9046 PPAR-related publications. The PPARgene database now contains 225 experimentally verified PPAR target genes, including 83 target genes for PPAR*α*, 83 target genes for PPAR*β*/*δ*, and 104 genes for PPAR*γ*. Forty genes were common targets of at least two PPAR subtypes. Detailed information including tissues, species, reference PubMed IDs, and hyperlinks to the original articles in PubMed database was also provided.

### 3.2. Generation of Logistic Regression Models to Predict PPAR Target Genes

We generated a logistic regression model to predict novel PPAR target genes. To train the logistic regression model, experimentally verified target genes were used as positive examples. Equal numbers of negative examples were obtained by random sampling from the background gene sets. Since the three PPAR subtypes bind to a conserved core sequence and share some common target genes [24], we currently did not distinguish subtypes in our prediction model.

Firstly, we generated the prediction model only based on* in silico* PPRE recognition using the standard position weight matrices (PWM) model [16]. Because functional PPREs were also found in downstream region of the TSS [9–12, 25], we scanned PPREs on both upstream and downstream regions. Genes were predicted as target genes or not according to the PWM score (PS). Fivefold cross validation was used to evaluate the performance of this model. As shown in Table 1, the median precision, recall, *F*1, and AUC were 0.57, 0.49, 0.52, and 0.59, respectively. The performance was poor, which may be due to a high number of false predictions of PPREs. It is reported that conservation in regulatory regions can be used to enhance the predictive specificity [17–19]. We next performed comparative genomic analysis to identify putative PPREs conserved in mouse and rat. Orthologous genes were then classified according to conserved PPRE score (CPS). As shown in Table 1, the median precision, recall, *F*1, and AUC were 0.61, 0.68, 0.64, and 0.68, respectively, which indicated a better performance.

**Figure 1:**
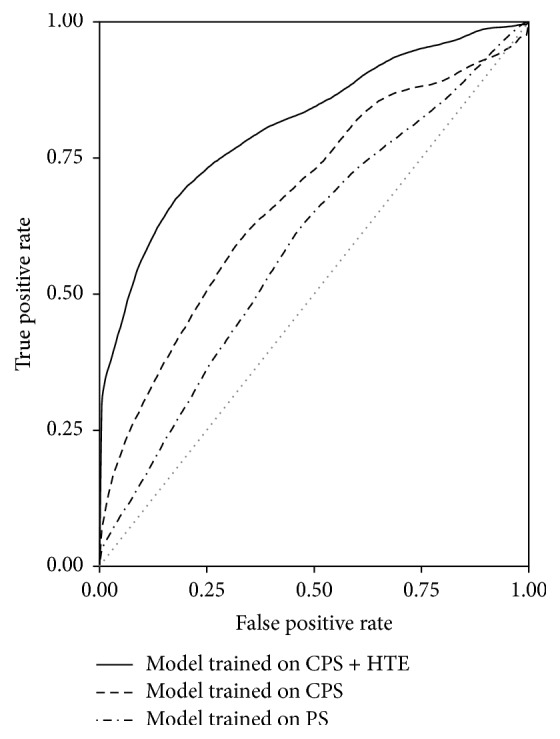
ROC curves for logistic regression models trained on different features. CPS: conserved PPRE score; HTE: high throughput evidence; PS: PPRE score.

Rather than* in silico* prediction of binding sites, experimental data sets provide direct evidence for gene regulation. Recently, high throughput technologies have produced a number of public available PPAR-relevant gene expression profiles. Thus, we collected PPAR-gain-of-function microarray data sets from the GEO database and extracted the supporting evidence for gene regulation. The logistic regression model was then generated based on a combination of conserved PPRE score and high throughput evidence. As shown in Table 1, the median precision, recall, *F*1, and AUC were 0.61, 0.68, 0.64, and 0.68. The performance was greatly improved. ROC curves of the prediction models also showed the improvement in performance (Figure 1).

### 3.3. Genome-Wide Prediction of PPAR Target Genes

**Figure 2:**
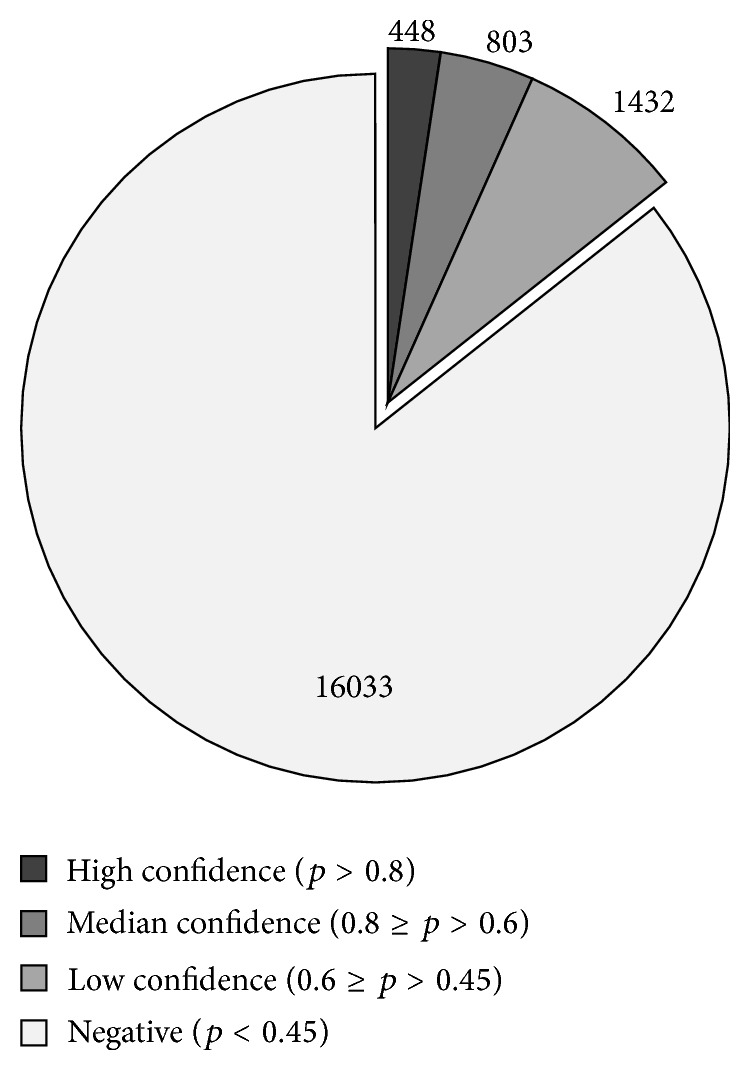
Number of predicted target genes in mouse genome. The predicted target genes were classified into 3 confidence levels according to the *p* value computed in the logistic regression model.

We predicted PPAR target genes from all 18,716 orthologous genes in mouse genome using the prediction model based on the combination of conserved PPRE score and high throughput evidence. We classified the predicted target genes into 3 confidence levels according to the *p* value (the probability of being a PPAR target gene) (Figure 2). In total, 2,683 genes with *p* > 0.45 were predicted as potential PPAR target genes, in which 448 genes were in the high-confidence category (*p* > 0.8), 803 genes were in the median-confidence category (0.8 ≥ *p* > 0.6), and 1432 genes were in the low-confidence (high-sensitivity) category (0.6 ≥ *p* > 0.45). Genes with *p* value ≤ 0.45 were predicted as negative. A complete list of the predicted PPAR target genes was available in the PPARgene website.

## 4. Querying the Database

The PPARgene database is composed of two modules: one is for querying experimentally verified target genes and the other is for querying computationally predicted target genes.

### 4.1. Experimentally Verified Target Genes

We provide users two ways to query the experimentally verified target genes. First, users can browse the results by selecting the PPAR subtype. PPARgene will return a table of matched entries. Users can also submit a specific gene symbol. The provided results contain the following items: PPAR subtype, gene symbol, species, tissue/cell types, regulation direction, and reference PubMed IDs.

### 4.2. Computationally Predicted Target Genes

**Figure 3:**
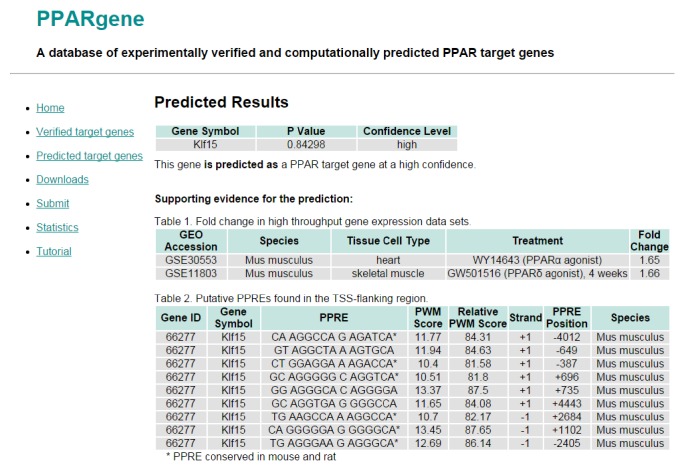
Predicted results of a query gene. High throughput gene expression data and putative PPREs were provided to support the prediction.

Users can retrieve the prediction results by querying the gene symbol. If the gene is predicted as a PPAR target gene, the query will return a *p* value with a confidence level. A larger *p* value means a higher confidence. High throughput gene expression data and putative PPREs were listed to support the prediction. For example, Klf15 was predicted as a PPAR target gene at a high confidence (Figure 3). The prediction was made based on the curated microarray data and identified PPREs. PPAR agonists WY14643 and GW501516 upregulated Klf15 expression in mouse heart and skeletal muscle tissues. In addition, 9 putative PPREs were found in the TSS flanking regions of mouse Klf15. Six of the 9 PPREs were also found in rat Klf15 and labeled with an asterisk. The PPRE in the +1102 has a highest PWM score (13.45). Thus, the logistic regression model integrated both the gene expression information and the highest PWM score of the PPRE to compute the probability value (*p*) as 0.84298, which placed Klf15 as a predicted target gene in the high-confidence category.

### 4.3. Downloadable Files

Users can download data sets of experimentally verified PPAR target genes as well as computationally predicted target genes. We also provide hyperlinks for downloading the high throughput experimental data sets curated in our prediction model.

## 5. Future Extensions

In this release of PPARgene, we have focused on curation and prediction of protein coding target genes. Recent studies demonstrated that PPARs regulate non-protein coding genes as well [26, 27]. Therefore, the future goal is to predict noncoding target genes of PPARs. We will also develop methods to predict target genes for each PPAR subtype. Experimentally supported PPAR target genes in the PPARgene database will be updated every 3 months.

## 6. Conclusion

In this study, we described PPARgene, a novel database of experimentally verified as well as computationally predicted PPAR target genes. By integrating* in silico* PPRE analysis with high throughput gene expression data, we developed an effective machine learning method to predict novel PPAR target genes in the mouse genome. We consider that PPARgene will be a useful tool for PPAR research.

## Figures and Tables

**Table 1:** Performances of logistic regression models trained on different features.

Features	Precision	Recall	*F*1	AUC
PS	0.57	0.49	0.52	0.59
CPS	0.61	0.68	0.64	0.68
CPS + HTE	0.83	0.59	0.69	0.82

PS: PPRE score; CPS: conserved PPRE score; HTE: high throughput evidence.
